# Comparative efficacy of the modified minimally invasive “Parachute Technique” vs. the intermuscular GAP Approach in proximal humeral fracture management: a prospective study

**DOI:** 10.3389/fsurg.2025.1541921

**Published:** 2025-06-16

**Authors:** Jia Jia Wei, Jun Li, Tao Chen, Hong Chi Yi, Wen Tao Zhao, Tian Tian, Jian Wei Wang

**Affiliations:** ^1^ The Affiliated Wuxi Hospital of Nanjing University of Chinese Medicine, Department of Orthopaedics, Wuxi, Jiangsu, China; ^2^ Yunnan Provincial Hospital of Traditional Chinese Medicine, Department of Spine Surgery, Kunming, Yunnan, China; ^3^ Changzhou TCM Hospital Affiliated to Nanjing University of Chinese Medicine, Department of Spine Surgery, Changzhou, China; ^4^ Yunnan University of Chinese Medicine, First Clinical Medical College, Kunming, Yunnan, China

**Keywords:** proximal humeral fractures, minimally invasive surgery, Parachute Technique, intermuscular Gap Approach, functional recovery

## Abstract

**Background:**

Proximal humeral fractures (PHFs) are common in elderly individuals, often resulting from osteoporosis and falls. Surgical intervention is required for displaced fractures to restore shoulder function. This study compares the Modified Minimally Invasive Parachute Technique and the Intermuscular Gap Approach in the management of displaced PHFs.

**Objective:**

To compare clinical outcomes, including surgical efficiency, complication rates, functional recovery, and radiographic healing, between the two surgical techniques.

**Methods:**

A total of 40 patients aged 60 or older with displaced two- or three-part PHFs were randomized into two groups (*n* = 20 per group). Primary outcomes included surgical duration, intraoperative blood loss, and postoperative drainage. Secondary outcomes included pain relief (Visual Analog Scale), shoulder function (Neer Shoulder Score), and fracture healing (Radiographic Union Scoring System, RUST).

**Results:**

The Parachute Technique group had significantly shorter operation times (97.25 ± 16.09 min vs. 119.75 ± 17.13 min, *p* < 0.001) and lower blood loss (99.00 ± 25.06 ml vs. 207.50 ± 44.47 ml, *p* < 0.001). Postoperative drainage was also significantly reduced in the Parachute Technique group (81.50 ± 13.48 ml vs. 119.00 ± 21.01 ml, *p* < 0.001). Functional recovery, assessed by the Neer Shoulder Score, was significantly better in the Parachute Technique group at 3, 6, and 12 months (*p* < 0.001). At 6 months, radiographic healing showed a trend towards better union in the Parachute Technique group (9.00 ± 0.73 vs. 8.60 ± 0.59, *p* = 0.072). Complication rates were similar between the two groups (*p* = 0.68).

**Conclusion:**

The Modified Minimally Invasive Parachute Technique offers superior surgical efficiency, reduced blood loss, and better functional outcomes compared to the Intermuscular Gap Approach, making it a favorable option for elderly patients with displaced PHFs.

## 1 Introduction

Proximal humeral fractures (PHFs) are among the most prevalent fractures in the elderly population, largely attributed to age-related osteoporosis and falls (1, 2). These fractures can lead to significant morbidity, decreased quality of life, and functional limitations if not managed optimally (3). Selecting an effective surgical approach is critical for ensuring proper anatomical restoration, pain relief, and functional recovery (4). While nonoperative management may suffice for minimally displaced fractures, surgical intervention is often warranted for complex or displaced fractures to restore shoulder mechanics and improve long-term outcomes (5, 6).

**Figure 1:**
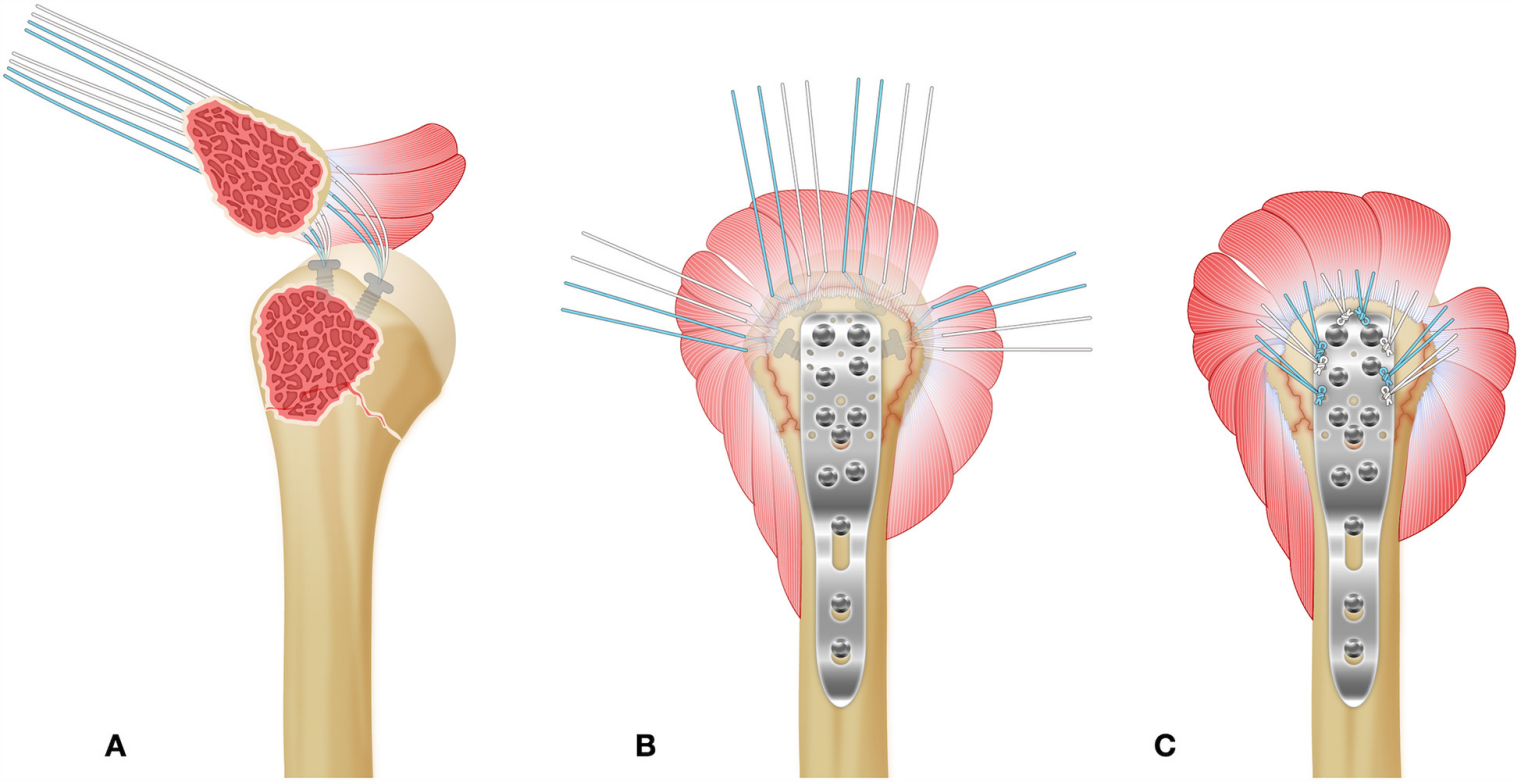
Surgical Technique for Proximal Humeral Fracture Management. **(A)** After fracture reduction, two anchors are placed at the proximal insertion of the rotator cuff tendons (shoulder cuff insertion point) to stabilize the fracture fragments. **(B)** Following fracture fixation, a proximal humeral plate is applied, and the suture tails from the anchors are passed through the rotator cuff tissue to further reinforce the fixation. **(C)** The suture tails are threaded through the side holes of the humeral plate and tied, tightening the rotator cuff tissue. This step consolidates fracture fixation and provides additional stabilization of the rotator cuff, ensuring enhanced healing and preventing displacement of the fracture fragments.

In recent years, advancements in surgical techniques have aimed to balance effective fixation with minimal soft tissue disruption (7). The minimally invasive parachute technique represents a modified approach designed to reduce surgical trauma and promote even stress distribution across the fracture site, with the intent of enhancing healing while reducing the risk of complications such as infection, hardware failure, and joint stiffness (8, 9) (Figure 1). In contrast, the intermuscular gap approach, utilizing the natural deltopectoral interval, enables direct access to the fracture with minimal disruption to surrounding soft tissues, thereby facilitating anatomical reduction and stable fixation (10, 11). Both techniques are widely used; however, data comparing their clinical efficacy remain limited, especially regarding postoperative function, complication rates, and patient-reported outcomes (12).

This study aims to fill this knowledge gap by conducting a prospective comparison of the modified minimally invasive parachute technique and the intermuscular gap approach in the management of proximal humeral fractures (13). Primary outcomes, including fracture union, pain levels, range of motion, and overall functional recovery, will be assessed to provide a comprehensive evaluation of each technique's advantages and limitations (14). This comparison seeks to offer evidence-based guidance to optimize surgical decision-making in proximal humeral fracture management (15).

## 2 Materials and methods

### 2.1 Study design

This study was approved by the medical ethics committee. All patient information is stored in the hospital database for research purposes. Inclusion Criteria: (1) Age: Patients aged 60 or older, typically affected by osteoporosis and falls; (2) Fracture Type: Acute two-part or three-part proximal humeral fractures (Neer classification) within 14 days of injury; (3) Fracture Displacement: Displaced fractures requiring surgical intervention for reduction and functional restoration; (4) Health Status: Medically stable, ASA score 1–3, with no severe comorbidities that could impair surgery or recovery; (5) Informed Consent: Capable of providing informed consent and willing to participate; (6) Surgical Eligibility: Suitable for either the Modified Minimally Invasive Parachute Technique or the Intermuscular Gap Approach. Exclusion Criteria: (1) Patients under 60, unless specific risk factors (e.g., severe osteoporosis) are present; (2) Fracture Type: Humeral shaft or distal fractures, four-part fractures, or fractures with significant articular in (1) volvement; (3) Infection: Active local or systemic infection, including wound infection or sepsis; (4) Comorbidities: Severe uncontrolled diabetes, cardiovascular disease, immunosuppression, or neurological disorders (e.g., stroke, Parkinson's); (5) Previous Surgery: Prior surgery on the same shoulder; (6) Psychosocial Factors: Cognitive impairment or psychiatric conditions preventing adherence to rehabilitation; (7) Pregnancy: Pregnant women, due to risks of anesthesia and radiation; (8) Anesthesia Contraindications: Conditions that contraindicate anesthesia

Initially, A total of 40 patients participated in this study, consisting of 17 males and 23 females, with an average age of 63∼80 years. All patients underwent either Modified Minimally Invasive “Parachute Technique” surgery or Intermuscular Gap Approach surgery.

Patients were divided into two groups based on the surgical method: 20 patients in the Parachute Technique group (including 7 cases of two-part fractures and 13 cases of three-part fractures, as classified by Neer) and 20 patients in the Intermuscular Gap Approach group (including 8 cases of two-part fractures and 12 cases of three-part fractures, as classified by Neer).

### 2.2 Surgical techniques

All patients underwent surgery performed by the same surgical team. Patients typically received either general anesthesia or regional anesthesia (brachial plexus block) to ensure they were pain-free and relaxed during the procedure. General anesthesia induces unconsciousness, while regional anesthesia numbs one side of the upper limb. The choice of anesthesia was based on the patient's condition and the surgeon's preference.

#### 2.2.1 The modified minimally invasive “Parachute Technique” group

The patient is positioned in the beach chair position under either brachial plexus block or general anesthesia via endotracheal intubation. Preoperative markings include the inferior border of the acromion and the course of the axillary nerve. The average distance from the axillary nerve to the top of the humeral head is approximately 6.1 cm (range: 5–7 cm). A longitudinal incision is made 1.5 cm inferior to the acromion, following the Distal Superior Acromial Split (DS) approach.

The incision begins proximally over the acromion and is extended distally for 3–5 cm, along the deltoid tuberosity. Blunt dissection is carried out along the muscle fibers of the deltoid. During dissection, the surgeon palpates the deep layers of the deltoid to assess the tension on the axillary nerve and ensures that the nerve and surrounding neurovascular structures are preserved. Any compression or transection of these structures should be avoided.

The deltoid muscle is further separated to expose the proximal humerus and the fracture site. Hematoma and entrapped soft tissue are removed, and fracture reduction is performed under direct or indirect visualization. A PHILOS plate is inserted along the humeral shaft, with temporary fixation using a Kirschner wire. Following satisfactory fracture reduction, screws are placed proximally, securing the plate in the inferior-medial aspect of the humeral head.

For fixation of the humeral head, anchor screws are inserted into the subchondral bone of the humeral head, typically via either the rotator cuff or the interval between the rotator cuff and the humeral head. These anchors are positioned at the superior aspect of the humeral head. Sutures are passed through the rotator cuff in a “parachute” configuration, with four parallel sutures being anchored to the plate holes.

**Figure 2:**
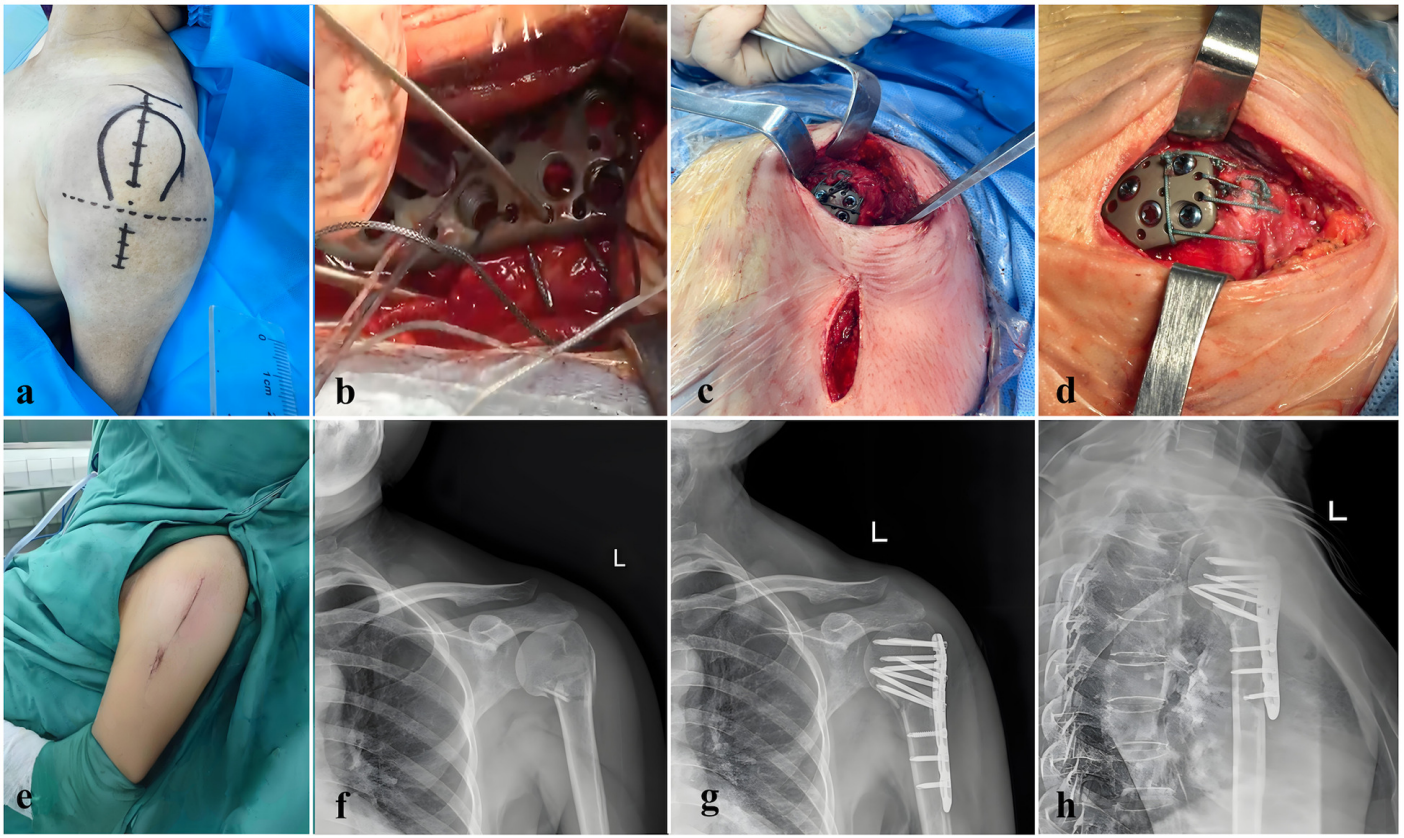
This figure illustrates key stages and outcomes in the surgical repair of a proximal humeral fracture using modified minimally invasive parachute technique. **(a)** Optimized incision design for the parachute technique in shoulder surgery, demonstrating a streamlined approach to access shoulder structures. This incision minimizes tissue damage and provides a stable foundation for precise suturing. **(b)** Suturing technique following rotator cuff repair, where sutures pass through a metal plate for temporary fixation. This step illustrates how both the fracture fragment and rotator cuff are temporarily secured, allowing precise alignment before final fixation. **(c)** Condition of upper and lower incisions after locking screw placement, showing effective fixation and alignment. The minimal soft tissue disruption observed here supports faster postoperative recovery and reduces the risk of complications. **(d)** Suture distribution after parachute stitching, ensuring uniform tension and stability across the repair site, which promotes optimal healing. The image shows a well-organized suture configuration, designed to withstand physiological loads on the shoulder. **(e)** Incision appearance with minimally invasive techniques, featuring small, precise entry points. The reduced incision size facilitates faster healing, minimizes scarring, and provides sufficient access for the procedure. **(f)** Preoperative anteroposterior x-ray showing a proximal humeral fracture, classified as a two-part fracture according to the Neer system, which guides preoperative planning. **(g)** Postoperative anteroposterior x-ray showing corrected alignment of the proximal humerus. The image confirms successful fracture reduction, with visible plate fixation indicating stable bone alignment. **(h)** Postoperative lateral x-ray, illustrating the final anatomical positioning of the proximal humerus and confirming the effectiveness of the repair. The lateral view allows precise verification of spatial alignment and fixation stability achieved through surgery.

Intraoperative fluoroscopy with a C-arm is used to confirm satisfactory reduction and fixation of the fracture. Proximal locking screws are placed for stable fixation. The plate length is determined based on its projection on the skin, and a small distal incision is made at the corresponding site. The position of the plate relative to the humeral shaft is verified by palpation, and locking screws are inserted using a drill through the locking sleeve, minimizing injury to the surrounding soft tissues. Temporary Kirschner wires are removed, and any associated rotator cuff tears are repaired. Hemostasis is achieved, and the wound is closed in layers (Figure 2).

#### 2.2.2 The intermuscular Gap Approach group

The procedure was performed under general anesthesia with the patient positioned supine or lateral, with the affected shoulder slightly elevated to optimize exposure. A 6–8 cm incision was made along the deltopectoral interval to access the proximal humerus while minimizing deltoid retraction and protecting the axillary nerve. After incision, the deltoid and pectoralis major were gently retracted to expose the long head of the biceps tendon, serving as an anatomical landmark for accessing the humeral head.

Under fluoroscopic guidance, the fracture was reduced by aligning the greater and lesser tuberosities, with temporary pins used for stabilization. A locking plate was positioned along the proximal humerus, featuring 4–6 proximal screw holes for fixation around the humeral head and tuberosities and 2–3 distal holes for securing the humeral shaft. Proximal screws were angled at 30°–45° and measured 30–50 mm in length, ensuring stable fixation without joint penetration. Distal screws were inserted perpendicularly (90°) to the shaft, with lengths of 20–35 mm, to provide stable distal fixation.

**Figure 3:**
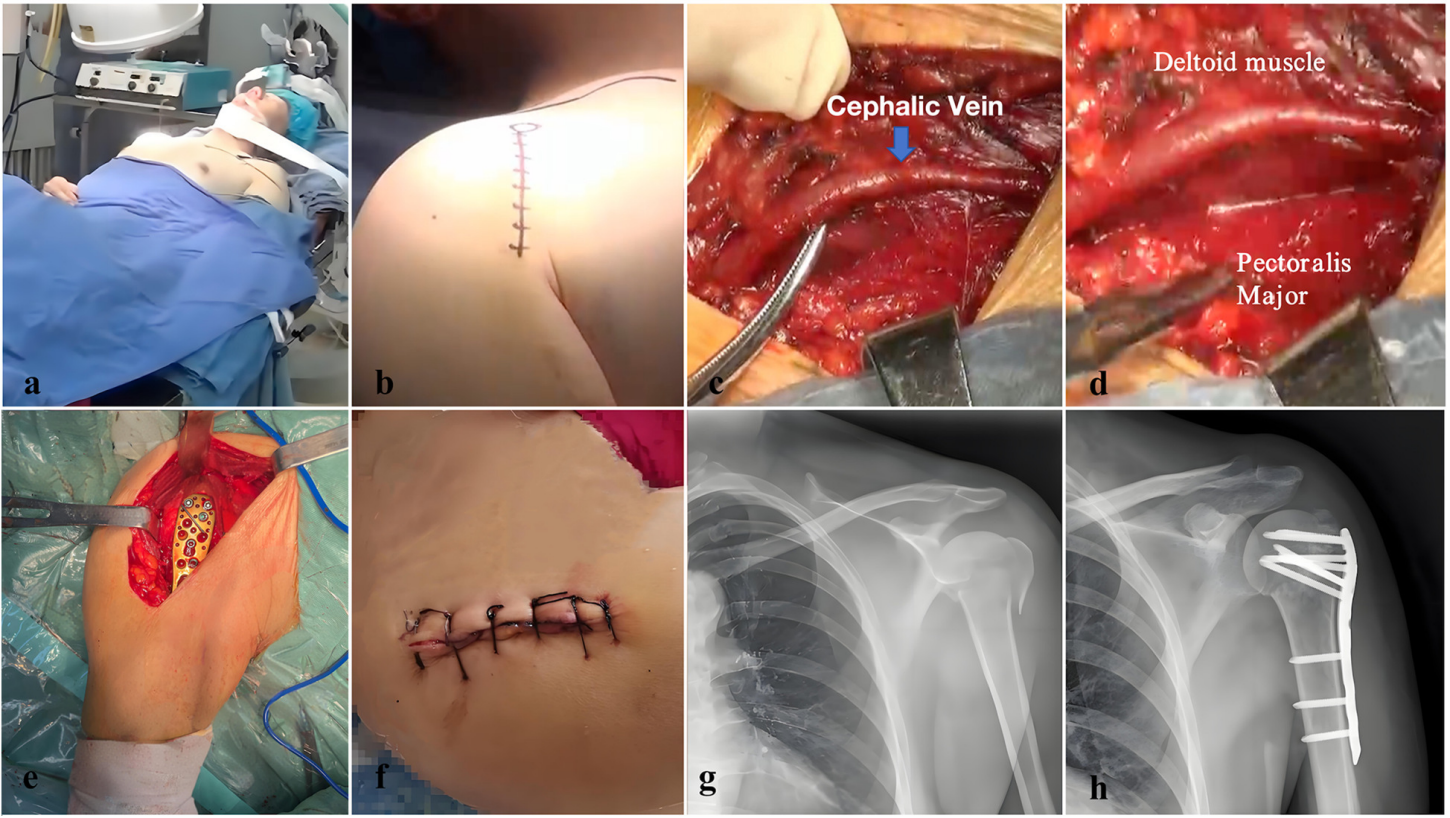
This figure illustrates the specific steps and surgical process of using an intramuscular interval approach to treat proximal humerus fractures. **(a)** Preoperative preparation with the patient under general anesthesia in the beach chair position, optimizing access to the shoulder joint. This position facilitates both fracture reduction and fixation during surgery. **(b)** Incision design using the deltopectoral approach, which provides direct access to the proximal humerus. The incision site is carefully planned to minimize soft tissue damage and allow efficient access to the fracture site. **(c)** Delicate separation and protection of the cephalic vein, a crucial step in preventing vascular injury during surgery. This meticulous dissection safeguards vascular integrity while maintaining a clear surgical field. **(d)** Exposure of the interval between the deltoid and pectoralis major muscles, leveraging this natural anatomical pathway to safely access the proximal humeral fracture. This approach minimizes disruption to muscle tissue, reducing postoperative complications and promoting a faster recovery. **(e)** After fracture reduction, fixation is achieved using a plate, with additional soft tissue suturing for reinforcement. This step secures the fracture and provides stability throughout the healing process. **(f)** Postoperative incision site, showing a well-healed wound that demonstrates the precision of minimally invasive surgery and the effectiveness of soft tissue preservation. **(g)** Preoperative anteroposterior x-ray showing the proximal humerus fracture, classified as a three-part fracture according to the Neer classification. **(h)** Postoperative anteroposterior x-ray displaying the aligned proximal humerus fracture, confirming successful reduction and fixation. The plate is visible, indicating stable fixation and optimal alignment of the fracture site.

The site was irrigated with saline, and soft tissues were closed in layers to promote healing and reduce infection risk. Postoperative care included shoulder immobilization and early passive motion exercises to maintain range of motion, with a gradual introduction of active movements to restore function and reduce stiffness (Figure 3).

### 2.3 Postoperative management

Postoperatively, the affected limb was immobilized with a shoulder sling to ensure protection and support. Prophylactic antibiotics were administered for 24 h, and the drainage tube was removed once output was sustained below 20 ml per 8-hour period. Patient-controlled analgesia was not employed; instead, analgesics were administered only if the pain score exceeded 5 on a standardized scale. All patients adhered to a uniform rehabilitation protocol, coordinated by licensed physical therapists from our institution's Department of Rehabilitation Medicine, with treatment sessions scheduled in a randomized manner.

### 2.4 Evaluation indicators

The following parameters were assessed to evaluate surgical and postoperative outcomes: surgical duration, intraoperative blood loss, and postoperative drainage volume at 24 h; preoperative and postoperative (days 1, 3, and 6) VAS pain scores; and postoperative laboratory markers including reduction in hemoglobin, leukocyte count, C-reactive protein (CRP), erythrocyte sedimentation rate (ESR), creatine kinase (CK), and creatine kinase-MB (CK-MB). Fracture healing was evaluated via anteroposterior and lateral shoulder x-rays taken at 3 and 6 months postoperatively, with scoring based on the Radiographic Union Scale for Tibial fractures (RUST), which assesses each of the four cortices (medial, lateral, anterior, and posterior) from 0 to 3 points per cortex, for a total score range of 4–12. Shoulder function was assessed using the Neer shoulder score at 3, 6, and 12 months postoperatively, evaluating pain, function, range of motion, and anatomical restoration, with higher scores indicating better functional recovery. Length of hospital stay and postoperative complications were also documented for both groups.

### 2.5 Statistical analysis

The collected data are analyzed with SPSS software (version 25.0). Measurement data are presented as mean ± standard deviation (x ± s), and comparisons between the Parachute Technique and Intermuscular Gap Approach groups are made using the Student's *t*-test. Categorical data are analyzed using the Chi-square test or Fisher's exact test. A *p*-value of less than 0.05 is considered statistically significant.

## 3 Results

A total of 40 patients with proximal humeral fractures were enrolled in this study, with 20 patients assigned to the Parachute Technique group and 20 patients to the Intermuscular Gap Approach group. The baseline demographic characteristics were comparable between the two groups, including sex (Parachute Technique: 8 males, 12 females; Intermuscular Gap Approach: 9 males, 11 females), age (Parachute Technique: 69.95 ± 4.15 years; Intermuscular Gap Approach: 71.95 ± 4.27 years, *p* = 0.058), and body mass index (BMI) (Parachute Technique: 25.04 ± 0.98 kg/m^2^; Intermuscular Gap Approach: 24.60 ± 1.46 kg/m^2^, *p* = 0.100). Fracture classifications were similar in both groups, with 7 patients in the Parachute Technique group and 8 patients in the Intermuscular Gap Approach group having two-part fractures, and 13 and 12 patients, respectively, having three-part fractures (Table 1).

**Table 1:** Comparison of clinical parameters between Parachute Technique and intermuscular Gap Approach groups.

Parameters	Parachute Technique group (*n* = 20)	Intermuscular Gap Approach group (*n* = 20)	*P*-value
Sex, *n*, (M/F)	8/12	9/11	0.25
Age, years, mean ± SD	69.95 ± 4.15	71.95 ± 4.27	0.058
BMI, kg/m^2^, mean ± SD	25.04 ± 0.98	24.60 ± 1.46	0.100
Neer classification:			
- two-part	7	8	
- three-part	13	12	
Hospitalization Duration, d, mean ± SD	7.50 ± 0.51	7.75 ± 0.64	0.096
Operation time,min, mean ± SD	97.25 ± 16.09	119.75 ± 17.13	0.000
Intraoperative Blood Loss, ml, mean ± SD	99.00 ± 25.06	207.50 ± 44.47	0.000
Postoperative Drainage Volume in 24 h,	81.50 ± 13.48	119.00 ± 21.01	0.000
ml, mean ± SD			
Reduction in Hemoglobin,	15.10 ± 2.86	24.60 ± 3.25	0.000
g/L, mean ± SD			
Postoperative Leukocyte count,*10^9/L,mean ± SD	12.20 ± 1.67	14.70 ± 1.98	0.000
Postoperative CRP levels, mg/L, mean ± SD	10.60 ± 2.98	14.65 ± 2.94	0.000
Postoperative ESR,	19.95 ± 6.62	29.05 ± 8.17	0.000
mm/h, mean ± SD			
Postoperative CK,	219.00 ± 61.03	339.50 ± 87.57	0.000
U/L, mean ± SD			
Postoperative CK-MB,	11.50 ± 2.89	16.30 ± 4.73	0.000
U/L, mean ± SD			
Complication:			0.68
Poor wound healing	0	1	
Infection	0	1	
Nerve injury	1	0	
Vascular injury	0	1	

BMI, body mass index; CRP, C-reactive protein; ESR, erythrocyte sedimentation rate; CK, creatine kinase; CK-MB, creatine kinase-MB.

*P*-value < 0.05 is considered statistically significant.

### 3.1 Surgical outcomes

The Parachute Technique group showed significantly better surgical outcomes across multiple parameters compared to the Intermuscular Gap Approach group. The operation time was significantly shorter in the Parachute Technique group (97.25 ± 16.09 min) compared to the Intermuscular Gap Approach group (119.75 ± 17.13 min, *p* < 0.001). Similarly, intraoperative blood loss was significantly lower in the Parachute Technique group (99.00 ± 25.06 ml) compared to the Intermuscular Gap Approach group (207.50 ± 44.47 ml, *p* < 0.001). The postoperative drainage volume during the first 24 h was also significantly reduced in the Parachute Technique group (81.50 ± 13.48 ml) compared to the Intermuscular Gap Approach group (119.00 ± 21.01 ml, *p* < 0.001) (Table 1).

The reduction in hemoglobin levels postoperatively was significantly smaller in the Parachute Technique group (15.10 ± 2.86 g/L) compared to the Intermuscular Gap Approach group (24.60 ± 3.25 g/L, *p* < 0.001), suggesting less blood loss during the procedure (Table 1).

Additionally, postoperative inflammatory markers, including the postoperative leukocyte count, C-reactive protein (CRP), erythrocyte sedimentation rate (ESR), creatine kinase (CK), and creatine kinase-MB (CK-MB), were all significantly lower in the Parachute Technique group, indicating a reduced inflammatory response postoperatively (all *p* < 0.001) (Table 1).

Regarding complications, the rates were similar between the two groups. In the Parachute Technique group, one patient experienced an axillary nerve injury, which was likely caused by intraoperative traction. The patient was treated postoperatively with neurotrophic medications, and full recovery occurred approximately three months after the surgery. In the Intermuscular Gap Approach group, there was one case each of poor wound healing, infection, and vascular injury. The vascular injury involved damage to the cephalic vein during the intermuscular gap approach, resulting in postoperative swelling of the affected limb. This was managed conservatively with elevation and compression, and the swelling resolved without further complications. One patient in the same group also experienced poor wound healing, which was attributed to the extensive soft tissue damage associated with the surgical approach. After routine wound care and dressing changes, the wound healed without any further complications. Additionally, one patient in the Intermuscular Gap Approach group developed a soft tissue infection, which was suspected to be related to inadequate drainage leading to a hematoma, subsequently causing infection. The infection was managed effectively by adjusting the antibiotic regimen, and the patient made a full recovery. However, these complications did not result in any statistically significant differences between the two groups (*p* = 0.68) (Table 1).

### 3.2 Functional outcomes

**Figure 4:**
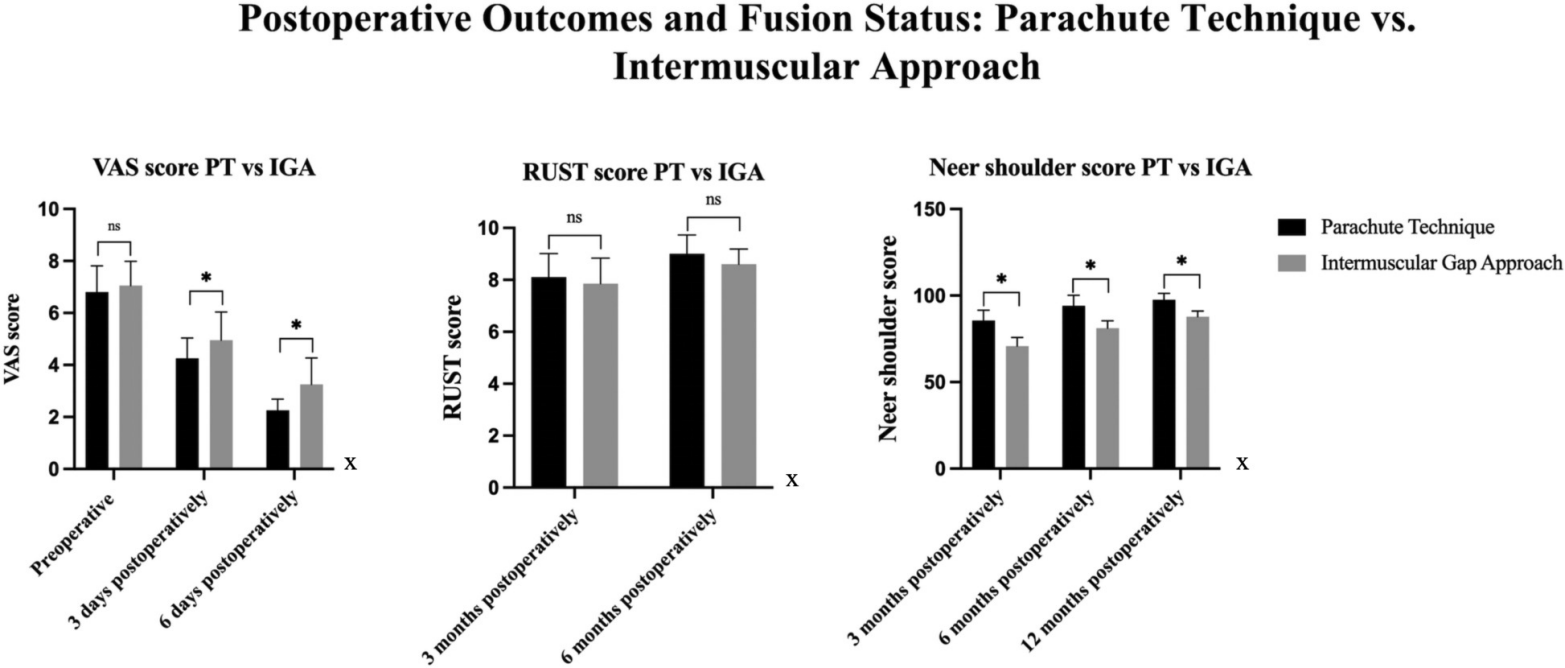
Comparison of VAS, RUST, and neer shoulder scores between PT and IGA groups. VAS Scores: Preoperatively, there was no significant difference between the PT and IGA groups. At 3 days postoperatively, a significant difference was observed, with the PT group exhibiting significantly lower VAS scores than the IGA group. This difference was also present at 6 days postoperatively, with the PT group showing lower VAS scores compared to the IGA group. RUST Scores: No significant difference was found between the PT and IGA groups at 3 months and 6 months postoperatively. Neer Shoulder Scores: At 3, 6, and 12 months postoperatively, significant differences were observed between the two groups, with the PT group showing significantly better outcomes than the IGA group at all time points.

Functional recovery was assessed using the Visual Analog Scale (VAS) for pain and the Neer Shoulder Score at multiple postoperative time points (Figure 4).

• Preoperative VAS scores were comparable between the two groups (Parachute Technique: 6.80 ± 1.01 vs. Intermuscular Gap Approach: 7.05 ± 0.94, *p* = 0.056).
• At 3 days postoperatively, the VAS score was significantly lower in the Parachute Technique group (4.25 ± 0.79) compared to the Intermuscular Gap Approach group (4.95 ± 1.09, *p* = 0.015).
• By 6 days postoperatively, the VAS score had decreased further in the Parachute Technique group (2.25 ± 0.44) compared to the Intermuscular Gap Approach group (3.25 ± 1.02, *p* < 0.001), suggesting faster pain relief in the Parachute Technique group.

In terms of shoulder function, the Neer Shoulder Score at 3 months postoperatively was significantly higher in the Parachute Technique group (85.50 ± 6.05) compared to the Intermuscular Gap Approach group (70.85 ± 5.01, *p* < 0.001). This difference remained significant at 6 months (Parachute Technique: 94.00 ± 6.19 vs. Intermuscular Gap Approach: 81.15 ± 4.31, *p* < 0.001) and at 12 months (Parachute Technique: 97.50 ± 3.81 vs. Intermuscular Gap Approach: 87.45 ± 3.36, *p* < 0.001), indicating better long-term functional outcomes in the Parachute Technique group (Table 2).

**Table 2:** Postoperative functional outcomes and fusion Status between Parachute Technique and intermuscular Gap Approach groups.

Parameters	Parachute Technique group (*n* = 20)	Intermuscular Gap Approach group (*n* = 20)	*P*-value
VAS score, mean ± SD			
- Preoperative	6.80 ± 1.01	7.05 ± 0.94	0.056
- Three days postoperatively	4.25 ± 0.79	4.95 ± 1.09	0.015
-Six days postoperatively	2.25 ± 0.44	3.25 ± 1.02	0.000
RUST score at 3 months postoperatively,mean ± SD	8.10 ± 0.91	7.85 ± 0.99	0.437
RUST score at 6 months postoperatively,mean ± SD	9.00 ± 0.73	8.60 ± 0.59	0.072
Neer shoulder score at 3 months postoperatively,	85.50 ± 6.05	70.85 ± 5.01	0.000
mean ± SD			
Neer shoulder score at 6 months postoperatively,	94.00 ± 6.19	81.15 ± 4.31	0.000
mean ± SD			
Neer shoulder score at 12 months postoperatively,	97.50 ± 3.81	87.45 ± 3.36	0.000
mean ± SD			

VAS, visual analog scale; RUST score, radiographic union scoring system score.

*P*-value < 0.05 is considered statistically significant.

### 3.3 Radiographic outcomes

Radiographic healing was evaluated using the Radiographic Union Scoring System (RUST) at 3 and 6 months postoperatively. At 3 months, there were no significant differences in RUST scores between the two groups (Parachute Technique: 8.10 ± 0.91 vs. Intermuscular Gap Approach: 7.85 ± 0.99, *p* = 0.437). However, at 6 months, the Parachute Technique group demonstrated a significantly higher RUST score (9.00 ± 0.73) compared to the Intermuscular Gap Approach group (8.60 ± 0.59, *p* = 0.072), reflecting a trend toward better radiographic union in the Parachute Technique group, though this difference was not statistically significant (Table 2).

### 3.4 Subgroup analysis by neer classification

#### 3.4.1 Two-part Fractures

**Figure 5:**
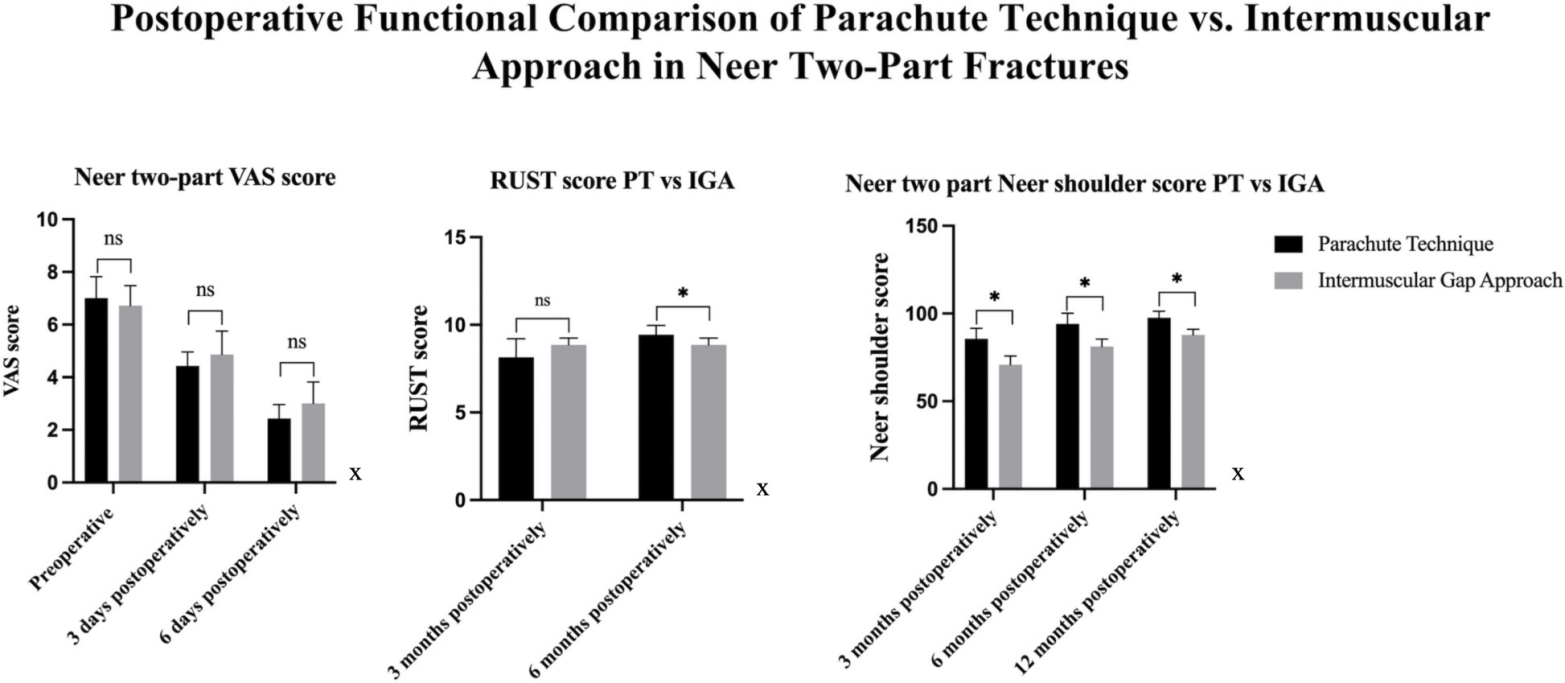
Comparison Of VAS, RUST, and neer shoulder scores between PT and IGA groups in neer Two-part fractures. VAS Scores: There were no significant differences in VAS scores between the PT and IGA groups at 3 days and 6 days postoperatively in both types of fractures. RUST Scores: At 3 months postoperatively, no significant difference was found between the two groups. However, at 6 months postoperatively, the PT group showed significantly better RUST scores than the IGA group. Neer Shoulder Scores: At 3 months, 6 months, and 12 months postoperatively, the PT group consistently demonstrated significantly superior Neer Shoulder scores compared to the IGA group.

• In patients with two-part fractures, functional outcomes were significantly better in the Parachute Technique group. At 12 months, the Neer Shoulder Score was significantly higher in the Parachute Technique group (98.57 ± 2.44) compared to the Intermuscular Gap Approach group (88.00 ± 0.76, *p* < 0.001). The RUST score at 6 months was also higher in the Parachute Technique group (9.43 ± 0.54 vs. 8.86 ± 0.38, *p* = 0.003), indicating better radiographic healing (Table 3) (Figure 5).

#### 3.4.2 Three-part fractures

**Figure 6:**
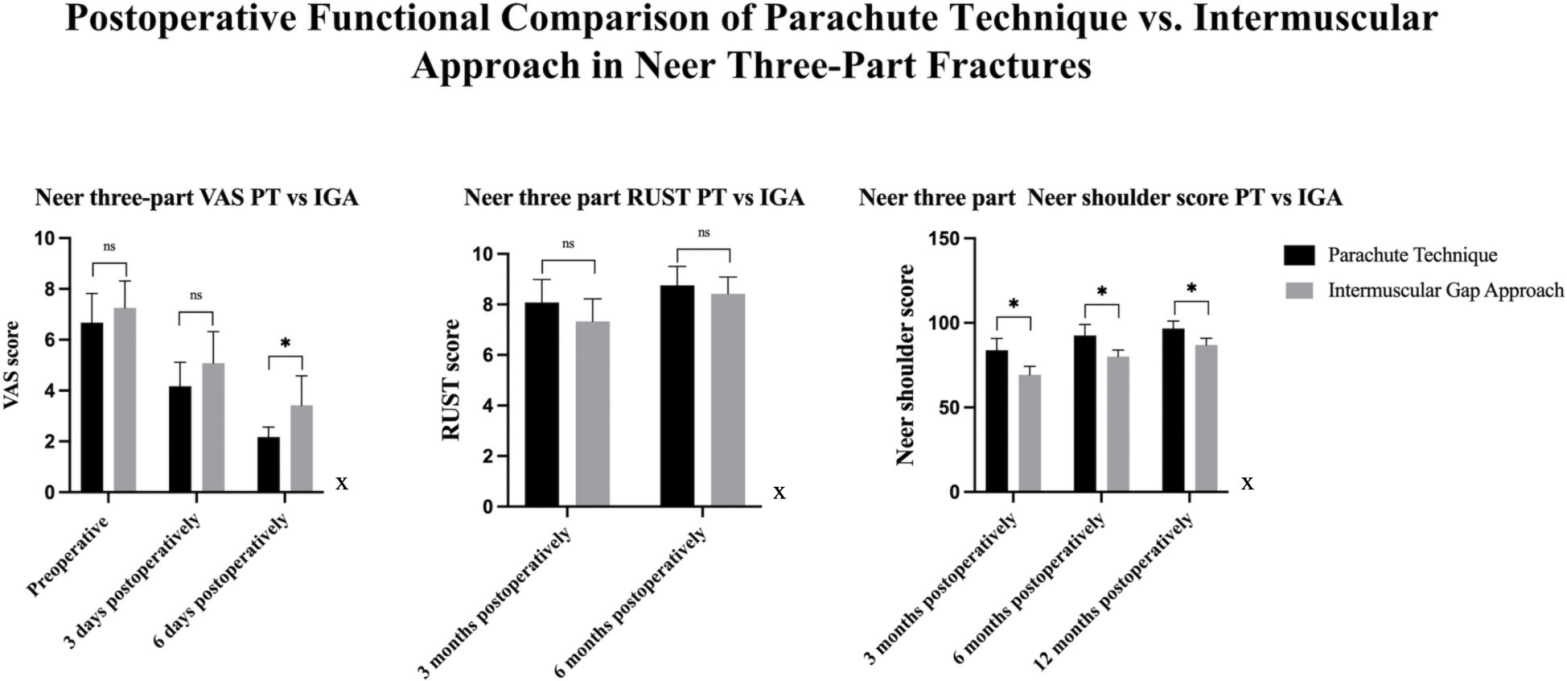
Comparison Of VAS, RUST, and neer shoulder scores between PT and IGA groups in three types of fractures. VAS Scores: There were no significant differences in VAS scores between the PT and IGA groups at 3 days postoperatively. However, at 6 days postoperatively, a significant difference was observed, with the PT group showing significantly lower VAS scores compared to the IGA group. RUST Scores: At 3 months and 6 months postoperatively, no significant differences were found between the PT and IGA groups. However, at 6 months, the PT group demonstrated significantly better RUST scores compared to the IGA group. Neer Shoulder Scores: At 3 months, 6 months, and 12 months postoperatively, the PT group showed consistently superior Neer Shoulder scores compared to the IGA group, with significant differences observed at all time points.

• In patients with three-part fractures, the Neer Shoulder Score at 12 months was significantly higher in the Parachute Technique group (96.67 ± 4.43) compared to the Intermuscular Gap Approach group (86.91 ± 4.03, *p* < 0.001). Similarly, the RUST score at 6 months was higher in the Parachute Technique group (8.75 ± 0.75 vs. 8.42 ± 0.67, *p* = 0.266), although this difference did not reach statistical significance (Table 3) (Figure 6).

The Parachute Technique demonstrated superior functional recovery compared to the Intermuscular Gap Approach for both two-part and three-part fractures. It also showed better radiographic healing at 6 months in two-part fractures. However, for three-part fractures, the difference in radiographic healing was not statistically significant.

**Table 3:** Comparison of postoperative functional outcomes between Parachute Technique and intermuscular Gap Approach groups based on neer classification.

Parameters	Parachute Technique group	Intermuscular Gap Approach group	*P*-value
Neer classification	*n* = 7	*n* = 8	
two-part			
VAS score, mean ± SD			
- Preoperative	7.00 ± 0.82	6.72 ± 0.76	0.522
- Three days postoperatively	4.43 ± 0.53	4.86 ± 0.89	0.356
- Six days postoperatively	2.43 ± 0.53	3.00 ± 0.82	0.172
RUST score at 3 months postoperatively, mean ± SD	8.14 ± 1.07	8.86 ± 0.38	0.094
RUST score at 6 months postoperatively, mean ± SD	9.43 ± 0.54	8.86 ± 0.38	0.003
Neer shoulder score at 3 months postoperatively, mean ± SD	87.86 ± 2.67	73.00 ± 4.73	0.000
Neer shoulder score at 6 months postoperatively, mean ± SD	95.72 ± 5.35	82.29 ± 4.82	0.000
Neer shoulder score at 12 months postoperatively, mean ± SD	98.57 ± 2.44	88.00 ± 0.76	0.000
Neer classification	*n* = 13	*n* = 12	
three-part			
VAS score, mean ± SD			
- Preoperative	6.67 ± 1.15	7.25 ± 1.06	0.131
- Three days postoperatively	4.17 ± 0.94	5.08 ± 1.24	0.067
- Six days postoperatively	2.17 ± 0.39	3.42 ± 1.16	0.004
RUST score at 3 months postoperatively, mean ± SD	8.08 ± 0.91	7.33 ± 0.89	0.121
RUST score at 6 months postoperatively, mean ± SD	8.75 ± 0.75	8.42 ± 0.67	0.266
Neer shoulder score at 3 months postoperatively, mean ± SD	83.75 ± 7.11	69.34 ± 4.98	0.000
Neer shoulder score at 6 months postoperatively, mean ± SD	92.50 ± 6.57	80.08 ± 3.89	0.000
Neer shoulder score at 12 months postoperatively, mean ± SD	96.67 ± 4.43	86.91 ± 4.03	0.000

VAS, visual analog scale; RUST score, radiographic union scoring system score.

*P*-value < 0.05 is considered statistically significant.

## 4 Discussion

Proximal humeral fractures (PHFs) are common, particularly in the elderly population, with osteoporosis and falls being the primary contributing factors (16). These fractures pose significant challenges in terms of surgical management, requiring optimal anatomical reduction, pain relief, and functional recovery (17). The choice of surgical technique is critical to achieving these goals, and this study compares two widely used approaches: the Modified Minimally Invasive Parachute Technique and the Intermuscular Gap Approach (18, 19).

### 4.1 Surgical outcomes and intraoperative considerations

Our study demonstrates that the Parachute Technique resulted in significantly better surgical outcomes, including shorter operation times and reduced intraoperative blood loss compared to the Intermuscular Gap Approach (20, 21). These findings align with previous studies suggesting that minimally invasive techniques, such as the Parachute Technique, offer benefits in terms of reduced surgical trauma (22, 23). By minimizing soft tissue dissection, this approach likely reduces the risk of complications such as blood loss, hematoma formation, and prolonged recovery (24). The Parachute Technique group's significantly lower postoperative drainage volumes further supports its advantages in minimizing soft tissue disruption (25).

In contrast, the Intermuscular Gap Approach, while providing excellent exposure to the fracture site, requires more extensive soft tissue dissection. This can lead to greater blood loss and longer operative times (26). These findings underscore the importance of choosing a surgical approach that balances adequate exposure with soft tissue preservation, as less invasive techniques can facilitate quicker recovery and reduced perioperative complications (27).

### 4.2 Postoperative inflammatory response

The Parachute Technique group also exhibited a significantly reduced postoperative inflammatory response, as evidenced by lower levels of C-reactive protein (CRP), erythrocyte sedimentation rate (ESR), and creatine kinase (CK) (28). This finding is consistent with previous literature, which suggests that less invasive surgical techniques lead to less systemic inflammation (29). The reduction in soft tissue manipulation during the Parachute Technique likely contributes to these lower levels of inflammation, potentially reducing the risk of postoperative complications such as wound infection, delayed healing, and joint stiffness (30).

While both groups showed elevated inflammatory markers postoperatively, the Parachute Technique's lower inflammatory response may contribute to faster recovery and better overall outcomes, particularly in terms of functional rehabilitation and healing (30). This reduced inflammatory response is one of the key advantages of minimally invasive techniques, allowing for more effective and efficient healing (28).

### 4.3 Functional and radiographic outcomes

The Parachute Technique demonstrated superior functional recovery compared to the Intermuscular Gap Approach, as assessed by the Neer Shoulder Score at 3, 6, and 12 months (20, 21). The Parachute Technique group achieved significantly better scores, indicating a faster recovery and better long-term functional outcomes (22). This aligns with the concept that minimizing surgical trauma, preserving soft tissues, and reducing postoperative inflammation can lead to improved functional recovery (23). Additionally, the lower Visual Analog Scale (VAS) pain scores at 3, 6, and 12 days postoperatively further support the superiority of the Parachute Technique in terms of pain relief and postoperative recovery (24).

Radiographically, the Parachute Technique also demonstrated a trend toward better healing, particularly in two-part fractures, where it showed significantly higher Radiographic Union Scale for Tibial fractures (RUST) scores at 6 months (25). While this difference was not statistically significant for three-part fractures, the trend suggests that the Parachute Technique may offer advantages in terms of radiographic healing, especially in simpler fractures (26). These results suggest that, in addition to better functional recovery, the Parachute Technique may promote more favorable fracture healing (27).

### 4.4 Subgroup analysis by fracture type

The analysis of two-part fractures revealed that the Parachute Technique resulted in significantly better functional and radiographic outcomes (28). These fractures, being less complex and comminuted, likely respond better to the less invasive approach offered by the Parachute Technique, which facilitates accurate reduction and stable fixation while minimizing soft tissue damage (29).

In the case of three-part fractures, while the Parachute Technique group demonstrated superior functional recovery, the difference in radiographic healing was not statistically significant (30). This could be due to the inherently more complex nature of three-part fractures, which may require more extensive surgical intervention and more direct manipulation of the fracture site (29). Nevertheless, the Parachute Technique's advantage in functional outcomes suggests that, despite the potentially more difficult fracture union, a less invasive approach can still provide meaningful improvements in postoperative function (30).

### 4.5 Complications and safety

Complication rates were similar between the two groups, with both groups experiencing manageable issues such as wound healing problems, infections, and nerve injuries (28). The Parachute Technique group had one case of axillary nerve injury, likely due to intraoperative traction. However, the injury was successfully treated with neurotrophic medications, and the patient made a full recovery within three months (29). In the Intermuscular Gap Approach group, complications included poor wound healing, infection, and vascular injury, all of which were resolved with appropriate management (30). The vascular injury involved damage to the cephalic vein, which caused postoperative swelling but was effectively treated with conservative measures (30).

These findings suggest that both techniques are safe when performed by experienced surgeons, but the Parachute Technique may result in fewer soft tissue-related complications, likely due to the smaller incision and reduced dissection (30). However, both approaches had manageable complication rates, with no serious adverse events or long-term sequelae (29).

While this study provides important insights into the comparative efficacy of the Parachute Technique and the Intermuscular Gap Approach, there are several limitations. First, the sample size was relatively small, and a longer follow-up period would be necessary to fully evaluate the long-term effects of each technique, particularly in terms of late complications, re-displacement of the fracture, and long-term function. Additionally, this study focused on relatively straightforward two-part and three-part fractures, and future research should investigate the efficacy of these techniques in more complex fractures, including four-part fractures or fractures with significant soft tissue injuries.

Further studies with larger sample sizes and extended follow-up periods are needed to confirm these results and assess the long-term durability and functional outcomes of the Parachute Technique in a broader patient population. It would also be valuable to explore the cost-effectiveness of these techniques, considering that minimally invasive approaches may result in shorter hospital stays and quicker recovery times.

## 5 Conclusion

The Modified Minimally Invasive Parachute Technique proved to be more effective than the Intermuscular Gap Approach for managing proximal humeral fractures. It resulted in shorter operation times, less blood loss, and reduced postoperative inflammation. Functional recovery was significantly better in the Parachute Technique group, particularly for two-part fractures. While radiographic healing was slightly better with the Parachute Technique, the difference was not significant for three-part fractures. Both techniques had similar complication rates, indicating that both are safe options when performed by experienced surgeons. Overall, the Parachute Technique offers a less invasive, effective approach with improved functional outcomes and a lower risk of complications. Further research with larger cohorts and longer follow-up is needed to confirm these findings.

## Data Availability

The original contributions presented in the study are included in the article/Supplementary Material, further inquiries can be directed to the corresponding author.

## Studies in humans and animals

We expressly declare that we comply with all relevant regulations, including the ARRIVE guidelines, the U.K. Animals (Scientific Procedures) Act, 1986 and associated guidelines, EU Directive 2010/63/EU for animal experiments, and the National Research Council's Guide for the Care and Use of Laboratory Animals.
